# Wheezing phenotypes and risk factors in early life: The ELFE cohort

**DOI:** 10.1371/journal.pone.0196711

**Published:** 2018-04-27

**Authors:** Souheil Hallit, Benedicte Leynaert, Marie Christine Delmas, Steffi Rocchi, Jacques De Blic, Christophe Marguet, Emeline Scherer, Marie Noelle Dufourg, Corinne Bois, Gabriel Reboux, Laurence Millon, Marie Aline Charles, Chantal Raherison

**Affiliations:** 1 U1219 INSERM, ISPED, Bordeaux University, Bordeaux, France; 2 INSERM U1152, Paris, France; 3 University Paris Dederot, Paris, France; 4 Sante Publique France, Saint-Maurice, France; 5 Parasitology Mycology Department, University Hospital, Besançon, France; 6 Chrono-environnement UMR CNRS 6249, Bourgogne Franche-Comté University, Besançon, France; 7 Pediatric Allergy and Pulmonology, CHU Necker Enfants Malades, Paris, France; 8 Pediatric and Pulmonology, CHU Rouen, France; 9 Unité Mixte INSERM-INED, Paris, France; Forschungszentrum Borstel Leibniz-Zentrum fur Medizin und Biowissenschaften, GERMANY

## Abstract

**Objective:**

Different phenotypes of wheezing have been described to date but not in early life. We aim to describe wheezing phenotypes between the ages of two months and one year, and assess risk factors associated with these wheezing phenotypes in a large birth cohort.

**Methods:**

We studied 18,041 infants from the ELFE (French Longitudinal Study of Children) birth cohort. Parents reported wheezing and respiratory symptoms at two and 12 months, and answered a complete questionnaire (exposure during pregnancy, parental allergy).

**Results:**

Children with no symptoms (controls) accounted for 77.2%, 2.1% had had wheezing at two months but no wheezing at one year (intermittent), 2.4% had persistent wheezing, while 18.3% had incident wheezing at one year. Comparing persistent wheezing to controls showed that having one sibling (ORa = 2.19) or 2 siblings (ORa = 2.23) compared to none, nocturnal cough (OR = 5.2), respiratory distress (OR = 4.1) and excess bronchial secretions (OR = 3.47) at two months, reflux in the child at 2 months (OR = 1.55), maternal history of asthma (OR = 1.46) and maternal smoking during pregnancy (OR = 1.57) were significantly associated with persistent wheezing. These same factors, along with cutaneous rash in the child at 2 months (OR = 1.13) and paternal history of asthma (OR = 1.32) were significantly associated with increased odds of incident wheezing. Having one sibling (ORa = 1.9) compared to none, nocturnal cough at 2 months (OR = 1.76) and excess bronchial secretions at 2 months (OR = 1.65) were significantly associated with persistent compared to intermittent wheezing.

**Conclusion:**

Respiratory symptoms (cough, respiratory distress, and excessive bronchial secretion) were significantly associated with a high risk of persistent wheezing at one year. Smoking exposure during pregnancy was also a risk factor for persistent and incident wheezing.

## Introduction

Asthma is one of the most common chronic diseases worldwide with an estimated 300 million individuals affected [1]. In clinical practice, asthma causes symptoms such as wheezing, shortness of breath, chest tightness, and coughing that vary over time in their occurrence, frequency, and intensity. However, a proper asthma diagnosis cannot be based on a single common feature [2, 3]. Currently, the intermittent symptoms of asthma are known to occur on a background of persistent airway inflammation and remodeling [4]. Asthma is no longer considered as just one disease but rather a multifaceted one, with multiple associated syndromes expressing different phenotypes [5]. Therefore, increasing knowledge of these phenotypes will provide better understanding of the disease in order to better assist and manage patients [5].

Approximately 25 to 30 percent of infants will have at least one episode of wheezing during their lifetime [6]. Children with persistent asthma may have reduced lung function, and some are at risk of accelerated decline in lung function in early adult life [7]. Irregularities in pulmonary function that are correlated with persistent wheezing become recognized during early childhood and continue into adult life, suggesting that early life exposures are critical in determining the onset and natural history of wheezing disorders [8–10]. The etiology of wheezing is usually difficult to ascertain, and wheezing is not specific for asthma and could be due to viral infection [11], or environmental exposure (such as smoking) [12]. The number of wheezing episodes in childhood seems to have an impact as outcomes are generally quite good in children with small number of wheezing episodes associated with viral illness [13].

The severity of symptoms has been correlated with a high frequency of wheezing episodes (usually more than three per year), resulting in the identification of frequent wheezing as a distinct phenotype [14–16]. Better understanding of the respiratory phenotypes in early childhood is of fundamental importance in addressing the risk factors for asthma and wheezing illnesses in children [8–10]. Four wheezing phenotypes (no wheezing, transient-early, late-onset, and persistent wheezing) were previously defined according to whether the child had reported wheezing during the first three years of life and whether they were still wheezing at age six [6]. Two British cohort studies used latent class analysis to identify distinct phenotypes underlying the observed heterogeneity in asthma symptoms during childhood [17, 18]. The Avon Longitudinal Study of Parents and Children (ALSPAC) identified six wheezing phenotypes in childhood from birth to seven years old [17]. However, it is unclear whether phenotypes identified by latent class analysis are comparable between birth cohorts observed in different areas or countries, particularly because the number and timing of measurements, definitions of wheezing, and population characteristics may differ between studies [19–21].

ELFE (French Longitudinal Study of Children) is the first large-scale French longitudinal birth cohort. The future follow-ups of the ELFE cohort will permit to derive phenotypes of wheezing throughout childhood and assess association with the development of asthma. However, to date, there is scarce available data on the distribution and change in respiratory symptoms at a very early age. Phenotypes of wheeze during the first year of life have not been studied before. The ELFE cohort comprises data on respiratory symptoms at two months and one year of age.

The aim of the present study was to characterize early wheezing phenotypes in infants, and identify risk factors associated with these phenotypes in a large French birth cohort.

## Methods

A representative random sample of mothers from maternity hospitals throughout France was established. The target population consisted of every one in 50 children born in 2011 in French maternity units [22]. After a written informed consent was obtained, medical records of the mother were consulted. The mother then answered a general questionnaire about the health of the baby and mental, social, family, and environmental exposures at age one. Follow-up is planned until age 18. The children were selected to represent all seasons of the year, having been born between April 1–4, June 27—July 4, September 27—October 4, and November 28—December 5, 2011. The exclusion criteria were: prematurity, childbirth under 18 years old, and no response to the questionnaire after two months. Mothers with multiple pregnancies with more than two children and those unable to give informed consent in one of the languages in which the information and consent forms were translated were also excluded.

**Fig 1:**
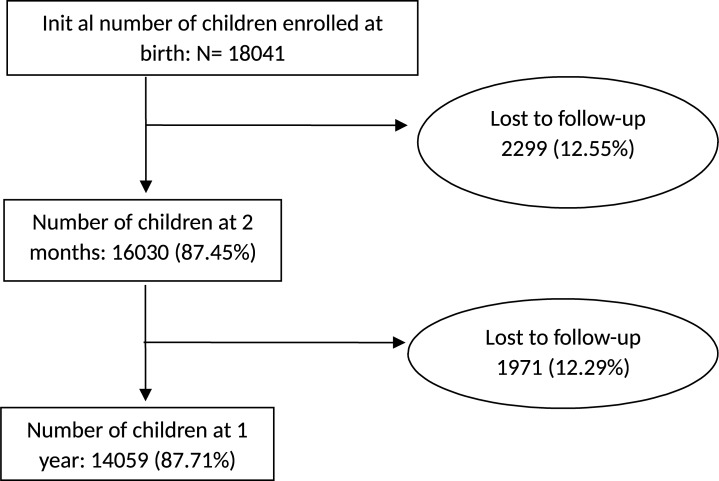
Flow chart.

Before the study began in 2011, a pilot study started in 2006–2007 in a small cohort (n = 470) to test the different parts of the study, and the questionnaires. Questions showing poor completion rates or that were not well understood by the participants were removed or corrected. Before the study, a training course was organized for the investigators (questionnaire, filters inside the questionnaire). Investigators traveled to maternity units and undertook the study in person with the parents. Initially, 18 322 children including 289 pairs of twins were enrolled in the study at birth. Among the 18,322 selected families who gave their consent, 281 eventually refused to participate, leaving 18,041 families in the study. The children were followed up at two months of age and then again at one year. At two months, trained investigators interviewed the parents in a face to face interview at home. When the child reached one year old, parents answered a standardized questionnaire through a telephone conversation conducted by specially trained professionals (phone-platforms currently involved in health studies in France, by the French institute of statistics (INSEE)). By the time of the phone interview, 4270 participants had been lost during the follow-up, leaving a total of 14059 children included in the present analysis (Fig 1).

### Wheezing phenotypes

Four groups were defined: non-wheezers (no current wheezing symptoms at both two months and one year old), intermittent (current wheezing symptoms at two months but no wheezing at one year), persistent (current wheezing at two months and at one year) and incident (no current wheezing at two months but wheezing at one year). Wheezing at two months was defined by a positive answer yes to the question “Does your child currently have wheezing or whistling in the chest? “[23]. Wheezing at one year was defined by a positive answer to the question “Does your child ever have wheezing or whistling in the chest? “

### Risk factors

The questionnaire assessed the sociodemographic characteristics (age, gender, region, number of rooms and number of persons living in the house, level of education for both parents), type of delivery, Apgar scores at one and five minutes respectively, mother’s previous pregnancies, and family history of asthma in parents and siblings of the child and other potential risk factors of asthma (heating system used inside the house, dampness inside the house, work done during pregnancy in the house, presence of pets in the house, child’s history of recurrent otitis, rash history of the child, bronchiolitis, bronchitis episodes, and breastfeeding). Information concerning parental allergies was recorded by questions about family history of eczema, asthma, eczema, and rhinitis (hay fever) for both parents and siblings. Finally, environmental exposure to toxic substances was considered: smoking during pregnancy, passive smoking, occupational and domestic pesticide exposure, and the use of cleaning products. Respiratory symptoms at two months were taken into account. The presence of coughing was ascertained by an affirmative answer to the question: “Has your child had a dry cough at night, apart from a cough associated with a cold or chest infection?” To know whether the child had respiratory problems, the following questions were used: “Has your child had any sign of respiratory distress?” and “At present does your child have any sign of respiratory distress?”. To investigate the presence of excess bronchial secretions, the following questions were used: “Has your child had an excess of bronchial secretion or phlegm?” and “At present are you noticing that your child has an excess of bronchial secretion or phlegm?”. A question was further asking if the child had ever been diagnosed with a bronchitis, and whether a doctor had prescribed medications, and a list of medications by class was proposed, to the parents.

### Ethical approval

Each parent gave written informed consent at the maternity unit. Every level of the project and each procedure was monitored by the National Council for Statistical Information (CNIS) and approved by the French Data Protection Authority (CNIL), in conjunction with the Consultative Committee for Data Processing in Health Research (CCTIRS), which assesses the scientific relevance of health projects, properly conducted from start to finish. Each parent retains the right to withdraw his/her child at any point during the study.

### Statistical analysis

Data analysis was performed by SPSS software, version 23. Two-sided statistical tests were used to test the association of each respiratory symptom at two months across all four wheezing groups; the Chi-2 test for dichotomous or multinomial qualitative variables, with Fisher’s exact test whenever appropriate. Regarding multivariate analysis, backward logistic regressions were performed by taking into account the variables in the bivariate analysis that showed a p-value <0.10 [24, 25];which included recommended covariates (such as maternal asthma, smoking, and educational level of the mother used as a proxy for socio-economical status). Educational level of the mother was categorized into 3 categories (low, intermediate and high level). Number of siblings was categorized into 0 (reference), 1, 2 and “3 or more” siblings. Several logistic regressions were conducted taking the dependent variables as follows: (1) intermittent vs persistent wheezing, (2) non-wheezers vs persistent wheezers and (3) non-wheezers vs incident wheezers.

## Results

During the two-month survey, 146 families quit the study and 29 children were born outside the dates of inclusion, reducing the total to 17,866 families. There were also 1295 non-responders, as well as 541 referent parents who did not answer the questions on the respiratory health of their children, leaving a final total of 16,030 children (Fig 1).

### Prevalence of wheezing and other respiratory symptoms at two months and one year

Prevalence of wheezing was 6.5 percent at two months and reached 27.5 percent at one year. The prevalence of nocturnal coughing increased from 25.5 percent to 67.5 percent between two months and one year, signs of respiratory distress increased from 29.1 percent to 38.8 percent and excess bronchial secretions from 34.7 percent to 65.1 percent respectively (Table 1).

**Table 1:** Prevalence of wheezing and other respiratory symptoms at two months and one year in ELFE cohort.

	Two months N = 15,896	One year N = 14,059
**Wheezing**	1026 (6.5%)	3871 (27.5%)
**Nocturnal cough**	4050 (25.5%)	9494 (67.5%)
**Respiratory distress sign**	4620 (29.1%)	5460 (38.8%)
**Excess bronchial secretion**	5519 (34.7%)	9155 (65.1%)

A significantly higher proportion of children with wheezing at two months and nocturnal coughing at two months were lost to follow-up, whereas a significantly higher proportion of children with maternal eczema and paternal hay fever were still participating at the one-year follow-up. There was no significant difference in patients lost to follow-up and those included in regards to gender, signs of respiratory distress or excess bronchial secretion in the child at two months, maternal history of asthma or hay-fever, and paternal history of asthma and eczema (Table 2).

**Table 2:** Comparison between children at one year and children lost to follow-up in ELFE cohort.

	Included (N = 14059)	Lost to follow-up Yes (N = 4270)	Total (N = 18329)	p-value
**Gender**				
**Male**	7112 (51%)	2169 (52.4%)	9281 (51.3%)	0.227
**Female**	6813 (48.9%)	1966 (47.5%)	8779 (48.5%)	
**Wheezing in child at two months**	830 (6.1%)	196 (8.7%)	1026 (6.5%)	<0.001
**Nocturnal cough in child at two months**	3437 (25.2%)	613 (27.3%)	613 (27.3%)	0.037
**Respiratory distress sign in child at two months**	3954 (29%)	666 (29.6%)	4620 (29.1%)	0.537
**Excess bronchial secretion in child at two months**	4729 (34.7%)	790 (35.1%)	5519 (34.7%)	0.653
**Maternal asthma**	1629 (12%)	239 (11%)	1868 (11.8%)	0.43
**Maternal eczema**	2063 (15.1%)	280 (12.9%)	2343 (14.8%)	0.01
**Maternal hay fever**	2758 (20.2%)	400 (18.5%)	3158 (20%)	0.156
**Paternal asthma**	1477 (12.8%)	129 (11.6%)	1606 (12.6%)	0.414
**Paternal eczema**	1049 (9.1%)	93 (8.3%)	1142 (9%)	0.717
**Paternal hay fever**	2256 (19.5%)	159 (14.2%)	2415 (19%)	<0.001
Mother’s educational level*				<0.001
Category 1	1997 (14.3%)	1379 (33.2%)	3376 (18.6%)	
Category 2	2632 (18.8%)	1164 (28%)	3796 (20.9%)	
Category 3	9361 (66.9%)	1608 (38.7%)	10969 (60.5%)	
**Age of the mother**	31.03 ± 4.81	29.41 ± 5.63		<0.001
**Number of siblings categories**				<0.001
None	6531 (46.5%)	2088 (48.9%)	8619 (47%)	
1	4971 (35.4%)	1276 (29.9%)	6247 (34.1%)	
2	1848 (13.1%)	540 (12.6%)	2388 (13%)	
3 or more	709 (5%)	366 (8.6%)	1075 (5.9%)	

* Category 1 includes illiteracy, primary and complementary levels of education; category 2 includes certificate of professional competence or professional qualifications, technological baccalaureate and secondary level of education; category 3 includes university and higher university degrees.

### Wheezing phenotypes at one year

Children with no symptoms (controls) accounted for 77.2 percent, 2.1 percent had had wheezing at two months but did not report wheezing at one year (intermittent), 2.4 percent had persistent wheezing, while 18.3 percent had incident wheezing at one year.

### Parental and infant factors associated with wheezing phenotypes

A significantly higher proportion of boys (59.5 percent) was observed among incident wheezers than among non wheezers (47.7%) (Table 3). Incident wheezers also showed a significantly higher proportion of children who were delivered by caesarean (19.2 percent) (vs. 18.3% in non-wheezers), and a higher proportion of children with at least one sibling (60 percent vs. 50% in non-wheezers). Having a nocturnal cough, signs of respiratory distress, and excess bronchial secretions at two months were associated with wheezing at one year (persistent wheezing). Persistent wheezers also comprise a significantly higher proportion of children with at least one sibling as compared to non wheezers. Maternal atopy (i.e. asthma, eczema, or hay fever) was also more frequent in persistent and incident wheezers. Smoking before and during pregnancy were significantly associated with persistent wheezing respectively (Tables 3 and 4). No significant difference was found between the different wheezing phenotypes and bronchitis, anti-asthma medication, and antibiotics taken by the child during the first year (data not shown).

**Table 3:** Infant risk factors associated with wheezing phenotypes in ELFE cohort.

Variable	Controls (N = 14151)	Intermittent (N = 393)	Persistent (N = 437)	Incident (N = 3348)	p-value
**Nocturnal cough at two months**	1954 (20.6%)	242 (61.6%)	338 (77.3%)	903 (27%)	<0.0001
**Respiratory distress signs at two months**	2343 (24.7%)	279 (71%)	360 (82.4%)	972 (29%)	<0.0001
**Excess bronchial secretions at two months**	2863 (30.2%)	294 (74.8%)	377 (86.3%)	1195 (35.7%)	<0.0001
**Gender**					<0.0001
Male	4661 (47.7%)	208 (53.6%)	266 (61.1%)	1977 (59.5%)	
Female	5119 (52.3%)	180 (46.4%)	169 (38.9%)	1345 (40.5%)	
**Presence of bronchiolitis in child at two months**	120 (5.5%)	43 (37.4%)	71 (43%)	41 (4.9%)	<0.0001
**Rash on the child's skin at two months**	1375 (14.5%)	74 (18.8%)	95 (21.7%)	559 (16.7%)	<0.0001
**Reflux in the child at 2 months**	1854 (19.6%)	96 (24.4%)	131 (30%)	768 (22.9%)	<0.0001

Results are expressed as frequency (percentage).

**Table 4:** Parental risk factors associated with wheezing phenotypes in ELFE cohort.

Variable	Controls (N = 14151)	Intermittent (N = 393)	Persistent (N = 437)	Incident (N = 3348)	p-value
**Maternal history of asthma**	1006 (10.6%)	52 (13.3%)	80 (18.3%)	491 (14.7%)	<0.0001
**Maternal history of eczema**	1369 (14.5%)	60 (15.3%)	69 (15.8%)	565 (16.9%)	0.01
**Maternal history of hay fever**	1827 (19.3%)	76 (19.4%)	112 (25.7%)	743 (22.3%)	<0.0001
**Paternal history of asthma**	956 (11.9%)	39 (12.7%)	47 (13.7%)	435 (15.1%)	<0.0001
**Maternal smoking during pregnancy**	1698 (17.4%)	85 (22%)	110 (25.5%)	657 (19.8%)	<0.0001
**Age of the mother at the child’s birth**	30.99 ± 4.88	30.97 ± 4.93	30.71 ± 4.57	31.19 ± 4.58	0.062
Maternal educational level**					<0.0001
Category 1	1407 (14.3%)	73 (18.7%)	71 (16.3%)	446 (13.4%)	
Category 2	1871 (19%)	86 (22%)	93 (21.4%)	582 (17.5%)	
Category 3	6555 (66.7%)	232 (59.3%)	271 (62.3%)	2303 (69.1%)	
**Number of siblings**					<0.0001
None	4891 (49.5%)	165 (42%)	129 (29.5%)	1346 (40.2%)	
1	3263 (33%)	146 (37.2%)	213 (48.7%)	1349 (40.3%)	
2	1237 (12.5%)	57 (14.5%)	74 (16.9%)	480 (14.3%)	
3 or more	490 (5%)	25 (6.4%)	21 (4.8%)	173 (5.2%)	
**Type of delivery**					0.016
Spontaneous labor	6577 (68.5%)	262 (68.2%)	306 (72.2%)	2283 (69.9%)	
Triggered childbirth	1269 (13.2%)	45 (11.7%)	42 (9.9%)	358 (11%)	
Caesarean	1753 (18.3%)	77 (20.1%)	76 (17.9%)	626 (19.2%)	
**Exposure to tobacco smoke inside the house (yes)**	1705 (17.3%)	76 (19.3%)	82 (18.8%)	537 (16%)	0.167
**Detergent use in house**	2793 (28.3%)	99 (25.2%)	127 (29.1%)	1002 (29.9%)	0.126
**Use of toiletry products of animal origin**	2223 (22.5%)	93 (23.7%)	90 (20.6%)	696 (20.8%)	0.146

Results are expressed as frequency (percentage) or mean ± Standard deviation.

** Category 1 includes illiteracy, primary and complementary levels of education; category 2 includes certificate of professional competence or professional qualifications, technological baccalaureate and secondary level of education; category 3 includes university and higher university degrees.

### Risk factors and wheezing phenotypes: Multivariate analysis

The results of a first backward logistic regression taking the intermittent vs persistent wheezers as the dependent variable, without entering the respiratory symptoms at 2 months as independent variables (Table 5), showed that having 1 sibling (adjusted odds-ratio ORa = 2.15; 95%CI 1.50–3.09) or 2 siblings (ORa = 1.75; CI 1.08–2.82) compared to none were significantly associated with persistent wheezing.

**Table 5:** Multivariable analysis.

**Logistic regression 1 taking intermittent vs persistent wheezers as the dependent variable (without the respiratory symptoms at 2 months as independent variables)**					
**Variable**	**p-value**		**ORa**	**Confidence Interval**	
Number of siblings categories (compared to no siblings)	<0.001				
1 sibling	<0.001		2.15	1.50	3.09
2 siblings	0.022		1.75	1.08	2.82
3 or more siblings	0.289		1.48	.71	3.10
**Logistic regression 2 taking non-wheezers vs persistent wheezers as the dependent variable (without the respiratory symptoms at 2 months as independent variables)**					
**Variable**	**p-value**		**ORa**	**Confidence Interval**	
Gender (males* vs females)	<0.001		.60	.48	.75
Number of siblings categories (compared to no siblings)	<0.001				
1 sibling	<0.001		3.18	2.43	4.17
2 siblings	<0.001		2.98	2.07	4.30
3 or more siblings	0.001		2.57	1.46	4.50
Cutaneous rash in the child at 2 months	<0.001		1.67	1.27	2.18
Reflux in the child at 2 months	<0.001		1.83	1.43	2.33
Maternal history of asthma	<0.001		1.64	1.21	2.23
Maternal smoking during pregnancy	<0.001		1.67	1.28	2.16
**Logistic regression 3 taking non-wheezers vs incident wheezers as the dependent variable (without the respiratory symptoms at 2 months as independent variables)**					
**Variable**	**p-value**	**ORa**		**Confidence Interval**	
Gender (males* vs females)	0.018	.61		.55	.66
Number of siblings categories (compared to no siblings)	0.03				
1 sibling	0.018	1.37		1.05	1.78
2 siblings	0.021	1.51		1.06	2.14
3 or more siblings	0.119	1.53		0.89	2.60
Reflux in the child at 2 months	0.019	1.38		1.05	1.80
Maternal history of asthma	0.044	1.42		1.01	1.97
Maternal smoking during pregnancy	0.039	1.35		1.01	1.79

* Reference group

-Variables entered in the models: maternal history of asthma, maternal history of eczema or hay fever, paternal history of asthma, maternal smoking during pregnancy, type of delivery, gender, number of siblings categories, rash on the child’s skin at 2 months, reflux in the child at 2 months, age of the mother at the child’s birth, educational level of the mother.

**Fig 2:**
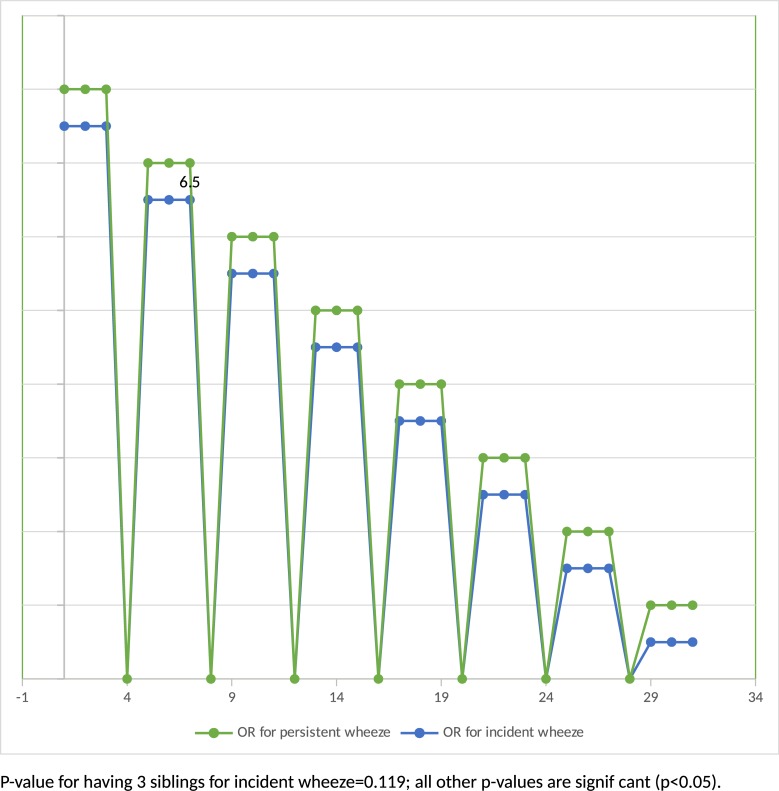
Factors associated with persistent and incident wheezing at one year compared to non-wheezers (odds-ratios from backward logistic regressions).

Fig 2 shows the odds ratios from the logistic regression for persistent wheezing and incident wheezing, as compared to non-wheezers. Female gender was associated with a decreased odds of both persistent wheezing and incident wheezing with OR of similar magnitude (Fig 2). Converserly, the presence of reflux in the child at 2 months, a maternal history of asthma and maternal smoking during pregnancy were significantly associated with an increased risk of both persistent and incident wheezing. Having 1, 2 or 3 or more siblings was a strong risk factor for persistent wheezing (with all OR>2.5) and was also associated with incident wheeze, although the association somewhat less strong. The presence of cutaneous rash in the child at 2 months was significantly associated with an increase in persistent wheezing.

When the respiratory symptoms at 2 months were entered in the model as independent variables (Table 6), the first backward logistic regression taking the intermittent vs persistent wheezers as the dependent variable, showed that the presence of a nocturnal cough in the child at 2 months (ORa = 1.76; CI 1.19–2.60) and excess bronchial secretions in the child at 2 months (ORa = 1.65; CI 1.04–2.59) were associated with an increase in the odds of persistent wheezing.

**Table 6:** Multivariable analysis.

**Logistic regression 1 taking intermittent vs persistent wheezers as the dependent variable (with the respiratory symptoms at 2 months as independent variables).**				
**Variable**	**p-value**	**ORa**	**Confidence Interval**	
Number of siblings categories (compared to no siblings)	.008			
1 sibling	0.001	1.90	1.31	2.76
2 siblings	0.121	1.47	0.90	2.41
3 or more siblings	0.69	1.16	0.55	2.45
Nocturnal cough in the child at 2 months	.005	1.76	1.19	2.60
Excess bronchial secretions in the child at 2 months	.031	1.65	1.04	2.59
**Logistic regression 2 taking non-wheezers vs persistent wheezers as the dependent variable.**				
**Variable**	**p-value**	**ORa**	**Confidence Interval**	
Gender (males* vs females)	<0.001	.63	.49	.81
Number of siblings categories (compared to no siblings)	<0.001			
1 sibling	<0.001	2.19	1.65	2.90
2 siblings	<0.001	2.23	1.53	3.24
3 or more siblings	0.03	1.92	1.06	3.46
Reflux in the child at 2 months	.001	1.55	1.18	2.02
Maternal history of asthma	.029	1.46	1.04	2.04
Maternal smoking during pregnancy	.002	1.57	1.18	2.08
Nocturnal cough in the child at 2 months	<0.001	5.20	3.91	6.92
Respiratory distress in the child at 2 months	<0.001	4.10	2.96	5.67
Excess bronchial secretions in the child at 2 months	<0.001	3.47	2.41	4.98
**Logistic regression 3 taking non-wheezers vs incident wheezers as the dependent variable.**				
**Variable**	**p-value**	**ORa**	**Confidence Interval**	
Gender (males* vs females)	<0.001	.61	.55	.66
Cutaneous rash in the child at 2 months	.037	1.13	1.01	1.28
Reflux in the child at 2 months	.001	1.20	1.08	1.34
Maternal history of asthma	<0.001	1.41	1.23	1.61
Paternal history of asthma	<0.001	1.32	1.16	1.49
Maternal smoking during pregnancy	.012	1.16	1.03	1.30
Maternal history of hay fever or eczema	.016	1.13	1.02	1.24
Nocturnal cough in the child at 2 months	<0.001	1.22	1.09	1.36
Excess bronchial secretions in the child at 2 months	.063	1.10	.99	1.21

* Reference group

-Variables entered in the models: maternal history of asthma, maternal history of eczema or hay fever, paternal history of asthma, maternal smoking during pregnancy, type of delivery, gender, number of siblings categories, rash on the child’s skin at 2 months, reflux in the child at 2 months, age of the mother at the child’s birth, educational level of the mother, nocturnal cough in the child at 2 months, respiratory distress in the child at 2 months, excess bronchial secretions in the child at 2 months.

A second backward logistic regression, taking non-wheezers vs persistent wheezers, with the respiratory symptoms at 2 months entered in the model as independent variables, showed that the presence of nocturnal cough in the child at 2 months (ORa = 5.2; CI 3.91–6.92), respiratory distress in the child at 2 months (ORa = 4.1; CI 2.96–5.67) and the excess bronchial secretions in the child at 2 months (ORa = 3.47; CI 2.41–4.98) were associated with an increase in the odds of persistent wheezing.

A third backward logistic regression, taking non-wheezers vs incident wheezers, with the respiratory symptoms at 2 months entered in the model as independent variables, showed that the presence of nocturnal cough in the child at 2 months (ORa = 1.22; CI 1.09–1.36) was associated with an increased odds of incident wheezing (Table 6).

## Discussion

In this large ELFE cohort, we found a high prevalence of respiratory symptoms in as early as two months. We also assessed different risk factors and respiratory symptoms associated with persistent wheezing at one year.

The percentage of children with respiratory symptoms at age two months and at one year old was higher compared to the Columbia Center for Children’s Environmental Health (CCCEH) (20.4 percent at age one) [26] and the ALSPAC [17] cohorts (26 percent at 18 months old). A nocturnal cough, signs of respiratory distress, and excess bronchial secretion symptoms continued to increase from two months old to one year. To our knowledge, no other cohort has evaluated these three symptoms with the development of respiratory symptoms at age one. Time will reveal the association between each of these symptoms and asthma at a later stage of these children’s lives. While all these symptoms are associated with an increased risk of having persistent wheeze at one year, it’s interesting to note also that nocturnal cough at the age of 2 months is also associated with an increased likelihood of incident wheeze at one year.

Nocturnal cough at two months, respiratory distress signs at two months, excess bronchial secretions at two months increased the likelihood of persistent and incident wheezing in the multivariate analysis. These symptoms could be due to allergenic inflammation, or inflammation related to environmental exposure to irritants in children exposed to a detrimental environment. These symptoms could also be due to viral infection, or to bacterial infection although endotoxin and other components of bacteria have been found to have a protective effect for asthma at an older age (hygiene hypothesis) [27]. Furthermore, the same respiratory symptoms may have different biological etiology.

Maternal smoking was associated with persistent wheezing (from two months to one year) and incident wheezing at one year, in agreement with what was found in previous cohorts [6]. The association between maternal smoking and transient early wheezing may be mediated, at least in part, by narrower airways in the children of women who smoke [6].

Parental asthma is thought to influence the risk of asthma in children [28]. However, hereditary patterns are multifaceted with many important questions remaining unanswered, such as heredity effects at different ages [6, 29], and the potential additional effect of having relatives with asthma. Our results indicate that a mother’s history of hay fever, eczema or asthma was associated with an increased risk of persistent wheezing in children, corroborating previous findings [30].

Furthermore, the increased number of siblings was associated with a decreased odds of persistent wheezing compared to intermittent wheezing, which might be explained by a higher risk of viral infection or contamination in children sharing their room with siblings. Indeed, previous findings suggest that the increased number of children puts the entire household at increased risk for infection with viruses that cause colds, flu, and other respiratory illnesses [31]. In addition, an increase in childhood asthma may predispose to severe RSV infection [32]. Meta-analyses have shown that delivery by caesarian section is associated with an increased risk of asthma in the offspring [33, 34], with a close and bilateral link between hospitalization for respiratory syncytial virus and asthma [35, 36].

The relationship between gastroesophageal reflux disease (GERD) and asthma in children has been investigated, although the nature of the association (if any) between them is unclear [37]. A clinical study showed that GERD is highly predominant in children with asthma, with estimations reaching as high as 80 percent, but nearly half of the children were asymptomatic; a finding in agreement with ours [38].

We have defined a priori three wheezing phenotypes, similar to those of Spycher et al. [39]. In the ALSPAC and PIAMA cohorts, six and five phenotypes were identified respectively [40]. A recent latent class analysis including respiratory symptoms (wheeze, night cough, and rhinitis) and atopy in children aged 18 months from the Pollution and Asthma Risk Infant Study identified a mild, a non-atopic severe and an atopic severe phenotype, respectively [41]. At age one, we also defined a transient wheezing phenotype (intermittent wheezing), consistent with the findings of Tucson [6]; since this phenotype is associated with having siblings, we can hypothesize that it is possibly linked to viral infections.

The strengths of our study include the early prospective documentation of respiratory symptoms and wheezing in a large nationally representative cohort with adjustment for key confounders including maternal asthma, exposure to smoking and maternal education and number of siblings. The use of parental reporting to identify respiratory symptoms and wheezing is a limitation, although we used an internationally standardized questionnaire. A selection bias is possible because of the refusal rate and the exclusion criteria. Indeed, a premature child or one from multiple pregnancies may exhibit respiratory or worse health at birth. In this case, the associations between phenotypes and severity criteria could be underestimated. In the French context, our inclusion criteria may have led to a selection bias with younger parents more often born abroad and having a lower occupational status being excluded from the study. Such women are more often single mothers and have a lower level of education than the parents in our sample. This may have led to an under-estimation of the number of less healthy children in our sample since children of such underprivileged mothers could have been under-represented in our sample. Furthermore, we cannot rule out an information bias since we used parent reporting. Another limitation of our study was the lack of viral measurement by PCR, which was not possible in this large population of newborn children, or even in a subsample of children because of the cost aspect.

However, the questionnaires were tested during pilot surveys, thus minimizing the information bias. Further, a follow-up of the ELFE cohort is underway, including respiratory symptoms, pulmonary function testing, and formal examination at age 10. These assessments will facilitate extended evaluation of the association between earlier wheezing phenotypes and subsequent respiratory health.

## Conclusion

We found a high prevalence of respiratory symptoms in early life with an increase in incident wheezing at age one. Other respiratory symptoms (cough, respiratory distress, and excessive bronchial secretion) were significantly associated with a higher risk of persistent wheezing. Smoking exposure during pregnancy remains a known risk factor of persistent wheezing. Longer follow-up with symptoms, lung function and atopy markers are needed to derive wheezing phenotypes throughout childhood and assess the relationships of these early-life symptoms with asthma onset.
