# Correction: Wheezing phenotypes and risk factors in early life: The ELFE cohort

**DOI:** 10.1371/journal.pone.0201863

**Published:** 2018-08-01

**Authors:** Souheil Hallit, Benedicte Leynaert, Marie Christine Delmas, Steffi Rocchi, Jacques De Blic, Christophe Marguet, Emeline Scherer, Marie Noelle Dufourg, Corinne Bois, Gabriel Reboux, Laurence Millon, Marie Aline Charles, Chantal Raherison

Fig 2 is incorrect. Please see the correct Fig 2 here.

**Fig 2:**
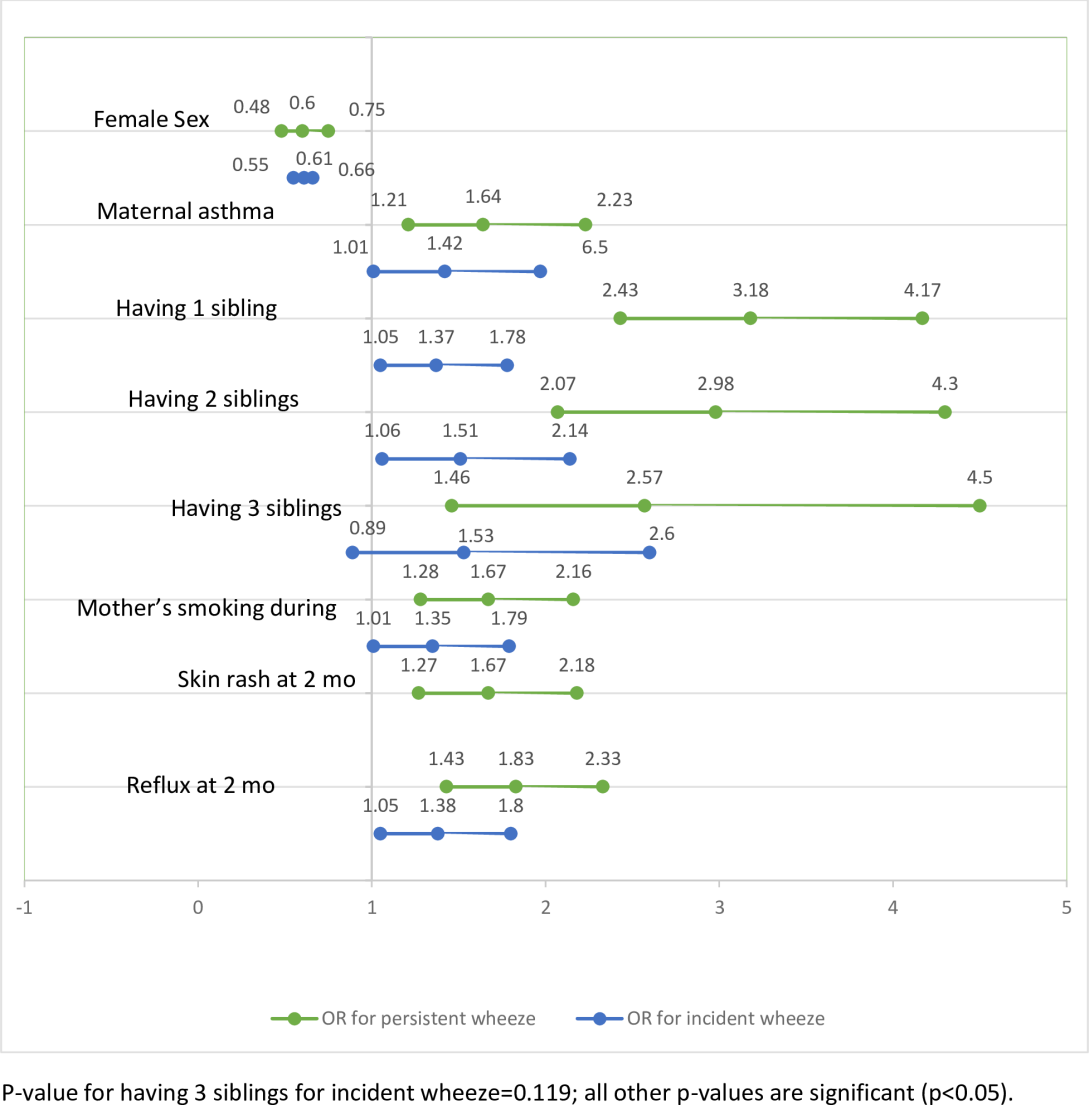
Factors associated with persistent and incident wheezing at one year compared to non-wheezers (odds-ratios from backward logistic regressions).
